# Monkeypox virus outbreak: can evolution guide us to new treatments or vaccines?

**DOI:** 10.1016/j.ebiom.2022.104221

**Published:** 2022-08-10

**Authors:** eBioMedicine

Until the beginning of 2022, human monkeypox was not a well-known disease among the general population. Sporadic cases of monkeypox occurred mainly in Central Africa and West Africa, where the virus is endemic. However, since May, 2022, cases of monkeypox have been confirmed outside of Central Africa and West Africa and the outbreak has been increasing at an alarming rate after the first case was confirmed in the UK on May 6^th^. The disease has also been spreading throughout other regions, such as Africa, the Americas, Eastern Mediterranean, and Western Pacific. According to OurWorldinData, as of July 25, there have been 16 313 confirmed cases of monkeypox worldwide in 75 countries. On July 23, WHO declared that monkeypox was a public health emergency. Monkeypox virus belongs to the *Orthopoxvirus* genus, the same genus as the virus that causes smallpox. Though there is no specific treatment for monkeypox, the genetic similarity between the viruses that cause the two diseases allows for the use of treatments and vaccines that are specific to smallpox.

African countries have been dealing with the monkeypox virus for at least 50 years, and scientists have been warning about a potential spread to other regions. Smallpox is highly infectious and it is transmitted by variola virus. After the eradication of smallpox in 1980, an epidemiological niche was created generating an opportunity for the spread of monkeypox virus. A conference report by Bunge and colleagues (2022) published in Vaccine showed that there has been an increase in cases since 1970, when the first human case of monkeypox was confirmed: from 48 confirmed cases in six African countries (between 1970 and 1979), increasing to 343 in 1980, and 520 in 1990. The first case outside the endemic region occurred in 2003 in the USA, with 47 cases caused by infected animals from Ghana that were sold as exotic pets. At that time, human-to-human transmission was not very common, with most cases related to animal reservoirs. The increase in cases of monkeypox could be related to, among other factors, climate change, armed conflicts in the region, rainforest exploitation, mobile populations, and waning herd immunity due to the eradication of smallpox.

In the 2022 outbreak, transmission has been distinct from previous African cases and human-to-human transmission is common. A phylogenomic analysis by Luna and colleagues (2022) published in Travel Medicine and Infectious Disease evaluated 337 genomes and showed that the current monkeypox virus has a specific monophyletic lineage compared with the viruses from previous outbreaks, with a mutational signature that could possibly favour transmission and dispersion. Before the 2022 monkeypox outbreak, monkeypox virus was classified into two clades: the Congo Basin Clade (clade 1), which is associated with severe symptoms and higher mortality rates, and the Central Africa clade (clade 2) which is associated with milder cases For the 2022 outbreak, the increase in transmission could be related to the genomic divergence from these two clades and a stochastic evolutionary event, which could explain the successful transmission and spread of monkeypox virus throughout different countries. Luna and colleagues proposed a third clade that is derived from clade 2 and is comprised of the hMPXV-1A clade, as well as the following lineages: A1, A1.1, A.2, and B.1, the latter emerging in Europe. Clade 3 is believed to have derived from cases of monkeypox from between 2018 and 2019. The 2018 cases occurred in Israel (one case) and the UK (two cases). In 2019, one case of monkeypox in Singapore and another in the UK were reported, which were both related to previous exposure. Genomes from the 2022 outbreak are still under investigation to assess whether they all belong to clade 3 or if a new clade is emerging. Transmission is characterised by sexual encounters and direct contact with body fluids, sores and scabs, and exposure to fomites; it can also occur by placenta transfer from mother to foetus. Though considered a mild disease, the main symptoms of monkeypox include fever, rash, and lymphadenopathy, as well as complications such as pneumonitis, encephalitis, sight-threatening keratitis, and secondary bacterial infections.

Smallpox vaccination ceased 40 years ago with the eradication of the virus, thus immunity against poxvirus is limited and susceptibility to the virus has increased over the years. Though smallpox was declared eradicated in 1980, antivirals were created for poxvirus treatment. A study by Grosenbach published at the New England Journal of Medicine investigated the efficacy of the poxvirus antiviral Tecovirimat in a monkeypox model, which led to survival rates of over 90% in non-human primates. Though the authors could not expose humans to smallpox intentionally, they conducted a randomised double-blind trial with humans to try to mimic the efficacious dose observed in the non-human primate model. Previous model studies predicted that a dose of 600 mg twice a day for 14 days would provide exposure in excess compared with animal models. Multiple-day dosing in human patients resulted in steady-drug accumulation that were approximately 50% (AUC_0-24h_) and 40% (C_max_) higher than the first dose, with steady-state levels seen at day 6. Thus, the Center for Disease Control and Prevention recommends Tecovirimat for those diagnosed with monkeypox virus. According to WHO´s immunisation guidelines, mass vaccination is not required, and both pre-exposure prophylaxis (PrEP) and post-exposure prophylaxis (PEP) are recommended depending on the situation. PrEP is recommended for those who have a high risk of exposure, and PEP for contact cases within 4 days of first exposure. Vaccines used are from second or third generation smallpox vaccines, JYNNEOS (also known as Imvamune or Imvanex) and ACAM2000 (IMVAMUNE). JYNNEOS has been recommended primarily because it is an attenuated, non-replicating live vaccine, from the Modified Vaccinia Ankara (MVA-BN) strain. Those who have immune deficiencies can thus benefit from this vaccine. ACAM2000 is not recommended for immunocompromised individuals as it can lead to serious disease. According to Fine and colleagues (1988) published at International Journal of Epidemiology, the vaccination efficacy is as high as 85%. However, these results date back when the first outbreaks occurred.

With the increase in cases of monkeypox around the world, vaccine shortage is expected. A one-dose shot has been used as strategy in some countries, such as Germany and the UK. Earl and colleagues (2008) published at PNAS administered one dose of MVA in macaques to assess protective immunity for monkeypox virus, antibodies were detected by ELISA after 7 days for MVA, and found a dominant response for IFN-ƴ. The authors found that a single dose of MVA could induce a rapid antibody and CD8+ and IFN-ƴ+ response. For protection against monkeypox virus, all vaccinated animals survived, providing evidence to show that a single dose is effective against infection. Pittman and colleagues (2019) published at the New England Journal of Medicine compared the efficacy of MVA vaccine with ACAM2000 for smallpox. They assigned two groups: one group that was assigned two doses of MVA and a third dose of ACAM2000, and a second group that was assigned to only a single dose of ACAM2000. Neutralising antibodies were higher for MVA than ACAM2000 group, and results indicated that one dose of MVA induced neutralising antibody titers similar to ACAM2000. For now, a single dose strategy might be one solution to the problem of low vaccine supply.

Vaccination for those at high risk of exposure to monkeypox should be a priority for policy makers, and low-income and middle-income countries should also vaccinate their high-risk populations. And though we have effective tools against monkeypox virus, real-world data from the 2022 outbreak is necessary to identify any shortcomings. Surveillance, epidemiology, detection, and control of the virus should be high priorities for the scientific community and public health officials. Communication with the population is also necessary to increase awareness and avoid the possibility of stigmatisation. At *eBioMedicine* we welcome robust studies to address the 2022 outbreak of monkeypox virus.

